# Early changes of CD4-positive lymphocytes and NK cells in patients with severe Gram-negative sepsis

**DOI:** 10.1186/cc5111

**Published:** 2006-11-27

**Authors:** Evangelos J Giamarellos-Bourboulis, Thomas Tsaganos, Ekaterini Spyridaki, Maria Mouktaroudi, Diamantis Plachouras, Ilia Vaki, Vassiliki Karagianni, Anastasia Antonopoulou, Vassiliki Veloni, Helen Giamarellou

**Affiliations:** 1 4^th ^Department of Internal Medicine, University of Athens, Medical School, 1 Rimini Str., Athens 124 62, Greece

## Abstract

**Introduction:**

Our aim was to define early changes of lymphocytes and of NK cells in severe sepsis and to correlate them with serum levels of soluble triggering receptor expressed on myeloid cells-1 (sTREM-1).

**Methods:**

Blood was sampled from 49 patients with proven highly suspected infection by Gram-negative pathogens, within 12 hours of the advent of severe sepsis, and was also sampled from six healthy volunteers. White blood cells were targeted with monoclonal antibodies and were analyzed by flow cytometry. The concentrations of sTREM-1 were estimated by ELISA.

**Results:**

The presence of CD3/CD4 cells was significantly lower (*P *< 0.0001) and that of NK cells significantly higher among patients with sepsis compared with controls (*P *= 0.011). The proportions (median ± standard error) of ANNEXIN-V/CD4/CD3-positive cells, of ANNEXIN-V/CD8/CD3-positive cells and of ANNEXIN-V/CD14-positive cells of the patient population were 7.41 ± 2.26%, 7.69 ± 3.42% and 1.96 ± 4.22%, respectively. Patients with NK cells >20% survived longer compared with those patients with NK cells ≤20% (*P *= 0.041), and patients with sTREM-1 concentrations >180 pg/ml survived longer compared with those patients with sTREM-1 concentrations ≤180 pg/ml (*P *= 0.042). A negative correlation was found between the percentages of ANNEXIN-V/CD4/CD3-positive cells and of CD3/CD4 cells (*r*_s _= -0.305, *P *= 0.049), and a positive correlation was found between the serum sTREM-1 concentration and the percentage of NK cells (*r*_s _= +0.395, *P *= 0.014). NK cells isolated from two healthy volunteers released sTREM-1 upon triggering with endotoxins.

**Conclusion:**

Early severe sepsis is characterized by CD4-lymphopenia and increased NK cells, providing a survival benefit for the septic patient at percentages >20%. The survival benefit resulting from elevated NK cells might be connected to elevated serum levels of sTREM-1.

## Introduction

Human studies in patients with sepsis have shown considerable changes in the subpopulations of lymphocytes [1], and particularly of those lymphocytes participating in adaptive immunity. These changes involve decreases of T-helper cells and of B lymphocytes. Data about the exact time point in the septic cascade where these changes occur are not available, however, although these data are of extreme importance since depletion of lymphocytes renders the septic hosts susceptible to further infectious insults.

Sparse data of either animal or human studies implicate a crucial role of new counterparts of the innate immune system in the pathogenesis of sepsis. These data comprise NK cells that are a subpopulation of lymphocytes behaving as cells of the innate immune system [2], as well as neutrophils and monocytes expressing the triggering receptor expressed on myeloid cells-1 receptor on their cell membranes in the event of human sepsis [3]. The soluble form of this receptor, namely soluble triggering receptor expressed on myeloid cells-1 (sTREM-1), is proposed to act as an anti-inflammatory mediator and to contribute to the transition from sepsis to septic shock [4,5].

Based on the latter evidence, the present study investigated whether changes of lymphocytes and NK cells occur early in severe sepsis. A cohort of patients with severe sepsis due to proven or highly suspected infection by Gram-negative bacteria was utilized. The use of this cohort stemmed from the necessity to study a population as homogeneous as possible for the type of antigenic stimulus. Changes of subpopulations of lymphocytes and of NK cells were also correlated to serum levels of sTREM-1.

## Patients and methods

### Study design

All patients hospitalized in the 4^th ^Department of Internal Medicine of the 'ATTIKON' University Hospital of Athens during the period November 2004 to January 2006 were delegates for the study. The protocol was approved by the Ethics Committee of the hospital and written informed consent was provided by the patients or their relatives.

Inclusion criteria were the concomitant presence of acute pyelonephritis, acute intra-abdominal infection or nosocomial pneumonia within the past 36 hours, and signs of severe sepsis within the past 12 hours. Exclusion criteria were neutropenia (≤500 neutrophils/mm^3^), HIV infection, oral intake of corticosteroids at a dose equal to or higher than 1 mg/kg equivalent prednisone for a period longer than one month, and administration of drotrecogin alpha prior enrolment.

Diagnosis of acute pyelonephritis was assigned to any patient with the following symptoms [6]: core temperature >38°C or <36°C, lumbar tenderness or radiological findings consistent with acute pyelonephritis, and pyuria defined as >10 white blood cells/high power field or +3 dipstick of urine for white cells.

Diagnosis of an intra-abdominal infection was made for any patient with the following symptoms [7]: core temperature >38°C or <36°C, white blood cells >12,000/μl, and pain on palpation or radiological findings consistent with an intra-abdominal infection.

Nosocomial pneumonia was diagnosed by the following criteria: the presence of symptoms at least 48 hours after hospital admission, provided that the infection was not under an incubation period prior to admission; a core temperature >38°C or <36°C; new or persistent consolidation in a lung X-ray scan; a sputum Gram strain with a predominance of Gram-negatives; and a modified clinical pulmonary infection score >5. The modified clinical pulmonary infection score was determined after individual scoring for each of the following parameters [8]: core temperature, 36.5–38.4°C = 0 points, 38.5–38.9°C = 1 point, and ≤36°C or ≥39°C = 2 points; white blood cell count, 4,000–11,000/μl = 0 points, <4,000 or >11,000/μl = 1 point, and >11,000 points and >10% bands = 2 points; pO_2_/FiO_2_, ≥240 or the presence of acute respiratory distress syndrome = 0 points, and <240 in the absence of acute respiratory distress syndrome = 2 points; diffuse shadows on lung X-ray scan = 1 point and localized shadow on lung X-ray scan = 2 points; and purulent tracheobronchial secretions = 2 points.

Severe sepsis was determined as the acute dysfunction of at least one organ, indicated by the acute presentation of at least one of the following [9]: acute respiratory distress syndrome, defined as any pO_2_/FiO_2 _<200; acute renal failure, defined as the production of <0.5 ml urine/kg body weight per hour for at least two hours provided that the negative fluid balance of the patient was corrected; metabolic acidosis, defined as any pH <7.30 or any base deficit >5 mEq/l and serum lactate at least more than twice the normal value; acute coagulopathy, defined as any platelet count <100,000/μl or an International Normalized Ratio (INR) >1.5; and acute cardiovascular failure, defined as systolic pressure <90 mmHg requiring the administration of inotropic agents for more than one hour provided the negative fluid balance of the patient was corrected.

Fifty-three patients were eligible for the study; four of these patients denied informed consent. A total of 49 patients were therefore enrolled.

Upon enrolment in the study, 10 ml blood was collected. Five milliliters were added to flasks for culture, another 3 ml were collected in an ethylenediamine tetraacetic acid-coated tube (Becton Dickinson, Cockeysville, MD, USA) for immunophenotyping, and the remaining blood was added in a sterile tube. After centrifugation, the serum was kept at -70°C until assayed. Flasks with blood were incubated for seven days. Identification of pathogens was performed by the API20E and the API20NE systems (bioMérieux, Paris, France). Enrolled patients were followed-up on a daily basis for a total of 28 days.

### Laboratory techniques

Red blood cells were lysed with ammonium chloride 1.0 mM in the whole-blood sample collected into ethylenediamine tetraacetic acid-coated tubes. White blood cells were washed three times with PBS (pH 7.2) (Merck, Darmstadt, Germany); the cells were subsequently incubated for 15 minutes in the dark with the monoclonal antibodies anti-CD3 and anti-CD19 and the protein ANNEXIN-V with the fluorocolor fluorescein isothiocyanate (emission 520 nm; Immunotech, Marseille, France), and with the monoclonal antibodies anti-CD4, anti-CD8, anti-CD14 and anti-CD(16+56) with the fluorocolor phycoerythrin (emission 550 nm; Immunotech) with or without the monoclonal antibody anti-CD3 with fluorocolor PC5 (emission 600 nm; Immunotech).

The following combinations were applied: anti-CD3/anti-CD4, anti-CD3/anti-CD8, anti-CD3/anti-CD(16+56), ANNEXIN-V/anti-CD4/anti-CD3, ANNEXIN-V/anti-CD8/anti-CD3 and ANNEXIN-V/antiCD14. Anti-CD19 was applied singularly. Cells staining positive for the above antibodies were analyzed after running through the EPICS XL/MSL flow cytometer (Beckman Coulter Co., Miami, FL, USA) with gating for lymphocytes or monocytes based on their characteristic FS/SS scattering. NK cells were defined as CD3-negative and CD(16+56)-positive cells.

Blood was also sampled from six healthy volunteers equally matched for age with the study population. IgG isotypic-negative controls with the fluorocolors fluorescein isothiocyanate and phycoerythrin (Immunotech) were applied before the start of analysis for each patient.

Estimation of sTREM-1 was performed by a home-made enzyme immunoassay. The capture antibody of sTREM-1 (R&D Inc., Minneapolis, MN, USA) was diluted to 4,000 ng/ml and distributed in a 96-well plate at a volume of 0.1 ml/well. After overnight incubation at 25°C, the wells were thoroughly washed with a 0.05% solution of Tween in PBS (Merck) (pH 7.2–7.4). Then 0.1 ml standard concentrations of sTREM-1 (15.1–4,000 pg/ml; R&D Inc.) or serum was added to the wells. After incubation for two hours, the wells were washed three times and 0.1 ml of one 400 ng/ml dilution of sTREM-1 detection antibody (R&D Inc.) was added per well. The plate was then incubated for two hours, and attached antibodies were signaled by streptavidin horseradish peroxidase. The concentrations of sTREM-1 in each well were estimated by the optical density detected at 450 nm after addition of one 1:1 solution of H_2_O_2_:tetramethylbenzidine as a substrate (R&D Inc.). All determinations were performed in duplicate; the inter-day variation of the assay was 5.23%.

### Isolation of NK cells

NK cells were isolated from two healthy volunteers, as already described [10]. Briefly, heparinized venous blood was layered over Ficoll Hypaque (Biochrom, Berlin, Germany) and was centrifuged. The buffy coat was washed three times with PBS (pH 7.2) (Merck), and was then diluted at a volume of 2 ml in RPMI 1640 (Biochrom) and incubated with 0.2 ml RosetteSep NK antibody cocktail (StemCell Technologies, Seattle, WA, USA) for one hour at room temperature with thorough mixing every ten minutes. The buffy coats were layered over Ficoll Hypaque and were centrifuged for 20 minutes at 1,200 × *g*. After centrifugation, any cells remaining over Ficoll Hypaque were collected and washed three times in PBS (pH 7.2). These cells were then stained with anti-CD3 fluorescein isothiocyanate and anti-CD(16+56) phycoerythrin, and were analyzed after running through the EPICS XL/MSL flow cytometer with the application of cells stained with IgG isotypic antibodies as negative controls. The collected cells were CD(16+56)-positive and CD3-negative at a purity greater than 90%.

NK cells were distributed into two wells of a 12-well plate with 2.4 ml RPMI 1640 enriched with 10% fetal bovine serum (Biochrom). Lipopolysaccharide of *Escherichia coli *O111: B4 (Sigma, St Louis, MO, USA) was added to the second well at a concentration of 10 ng/ml. The experiment was run in duplicate for each volunteer. After incubation for 18 hours at 37°C in 5% CO_2_, the content of each well was collected and centrifuged. sTREM-1 was estimated in the supernatants, as described above.

### Statistical analysis

Results are expressed as the median and interquartile range, but those of cell cultures expressed as the means and standard deviation. Comparisons between patients and healthy controls were performed by the Mann–Whitney *U *test. Statistical correlations were performed after assessment of the nonparametric Spearman coefficient (*r*_s_).

The time of survival was estimated after Kaplan-Meier analysis; patients were divided into two groups based on serum levels of sTREM-1 and on the percentage of NK cells. The patients were categorized into those with serum sTREM-1 ≤180 pg/ml and serum sTREM-1 >180 pg/ml, as proposed elsewhere [11]. After scattering of individual values of NK cells for survivors and nonsurvivors, the patients were also divided into those with NK ≤20% and NK >20%. Comparisons between these subgroups for survival were performed by log-rank tests.

Comparisons of qualitative data were performed according to Fischer's test. *P *< 0.05 was considered statistically significant.

## Results

Clinical characteristics of the patients enrolled in the study are presented in Table 1. Subpopulations of lymphocytes of patients compared with healthy controls are presented in Table 2. CD3/CD4 cells were significantly lower in early severe sepsis patients compared with controls (*P *< 0.0001). NK cells were significantly higher in early severe sepsis patients compared with controls (*P *= 0.011).

The median percentage of ANNEXIN-V/CD4/CD3-positive cells was 7.41%, with a 25th percentile of 1.70% and a 75th percentile of 19.81%. The median percentage of ANNEXIN-V/CD8/CD3-positive cells was 7.69%, with a 25th percentile of 2.13% and a 75th percentile of 17.00%. The median percentage of ANNEXIN-V/CD14-positive cells was 1.96, with a 25th percentile of 0.00% and a 75th percentile of 6.54%.

**Figure 1:**
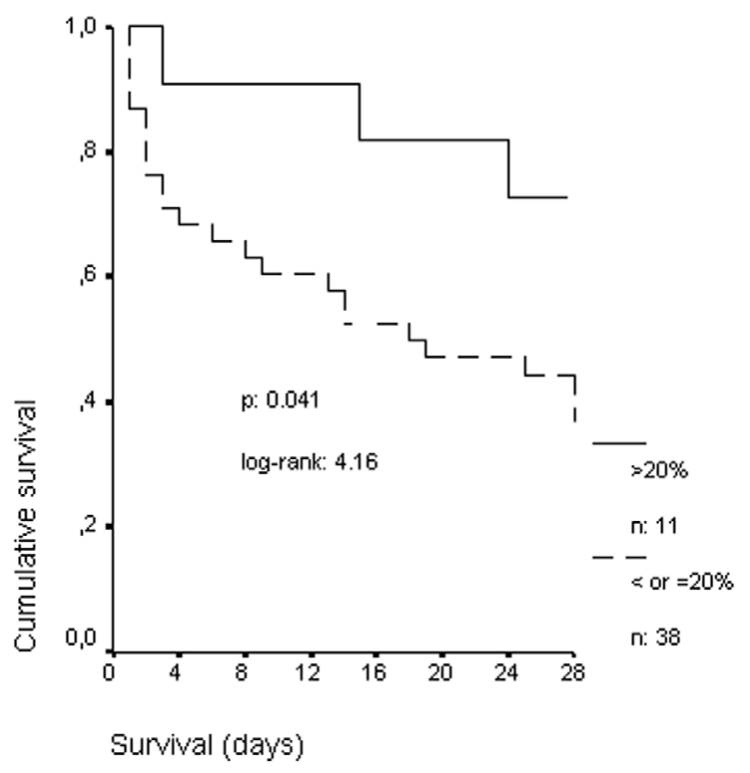
Survival curves of patients relative to the presence of NK cells.

The survival curves of patients in relation to the percentage of NK cells are shown in Figure 1. Patients with NK cells >20% survived longer compared with those patients with NK cells ≤20% (*P *= 0.041). Twenty-four out of 38 patients with NK cells ≤20% died (63.2%), compared with three out of 11 patients with NK cells >20% (27.3%, *P *= 0.046).

**Figure 2:**
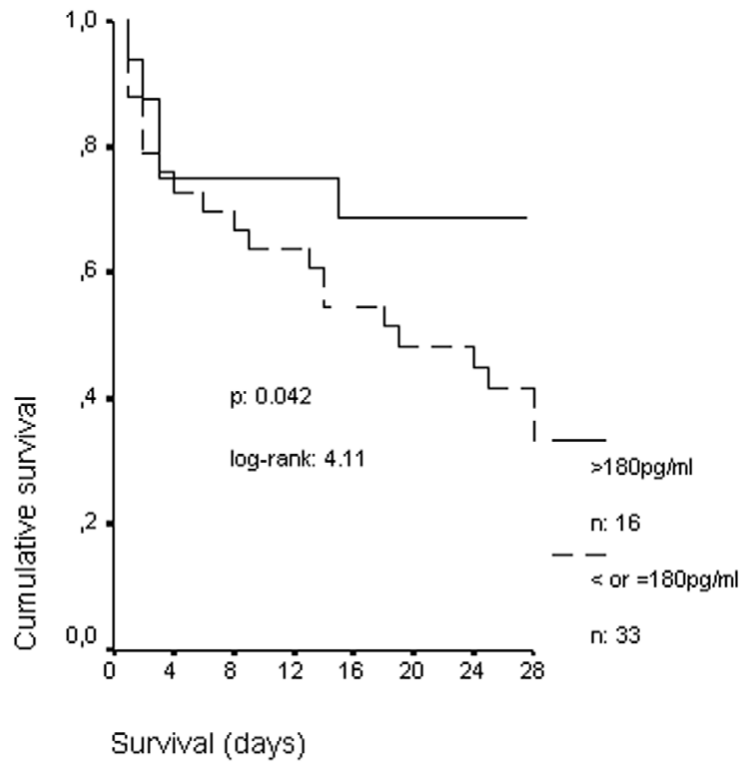
Survival curves of patients relative to serum soluble triggering receptor expressed on myeloid cells-1.

The survival curves of patients in relation to serum sTREM-1 levels are shown in Figure 2. Patients with sTREM-1 >180 pg/ml survived longer compared with those patients with sTREM-1 ≤180 pg/ml (*P *= 0.042). Twenty-two out of 33 patients with sTREM-1 ≤180 pg/ml died (66.7%), compared with five out of 16 patients with sTREM-1 >180 pg/ml (31.3%, *P *= 0.032).

A negative correlation was found between the percentages of ANNEXIN-V/CD4/CD3-positive cells and of CD3/CD4 cells (*r*_s _= -0.305, *P *= 0.049). A positive correlation was also found between serum sTREM-1 and the percentage of NK cells (*r*_s _= +0.395, *P *= 0.014).

**Figure 3:**
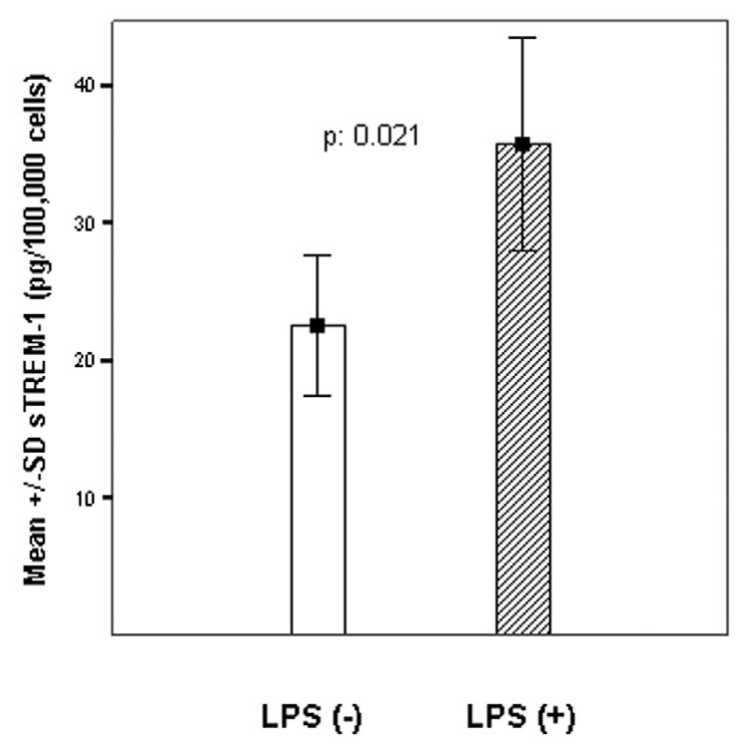
Release of soluble triggering receptor expressed on myeloid cells-1 by NK cells.

The concentrations of sTREM-1 released by NK cells of healthy volunteers and their modification after triggering by lipopolysaccharide are shown in Figure 3.

## Discussion

The identification of early changes taking place in the clinical setting of a septic host is of prime importance in order to understand the underlying pathogenesis. The majority of clinical studies do not provide evidence about the alterations of the immune responses within a short time frame after occurrence of organ failure. The lack of knowledge of early changes responsible for the transition from one stage of sepsis to another has been proposed as one reason responsible for the failure of numerous trials of immunointervention in sepsis [12].

The present study was designed to provide information about the early changes of adaptive immune responses and of NK cells occurring within the first 12 hours after advent of severe sepsis. Moreover, the entire population became septic because of the same antigenic stimulus – one Gram-negative pathogen. This was achieved by enrolling patients with infections clinically thought to be caused by Gram-negative bacteria or having been proved with a microbiological documentation of their infection being solely of Gram-negative origin (Table 1). The need for enrolment of patients with Gram-negative severe infections derived from the high frequency of Gram-negative bacteria as pathogens [13]. Findings of the study were correlated to serum levels of sTREM-1, which is a novel anti-inflammatory mediator of prime importance for the transition from severe sepsis to septic shock [4,5].

The results revealed two major early changes in the subpopulations of mononuclear cells of the study population. The first finding consists of a considerable decrease of CD3/CD4 lymphocytes (Figure 1). This depletion of CD4-positive lymphocytes resulted from cell apoptosis since the percentage of total CD3/CD4 cells was negatively correlated to the percentage of apoptotic CD3/CD4 cells. CD4-lymphopenia as a consequence of apoptosis is a well-described finding in septic patients [1,13-15]. The presented median rate of apoptosis of 7.41% CD4-positive cells is within the range reported elsewhere for septic patients [16]. The present study is the first to report the very early occurrence of CD4-lymphopenia in severe sepsis. The applied flow cytometric analysis of apoptosis did not use propidium iodine staining positive for necrotic cells. Application of the analysis was difficult due to the application of a triple combination of fluorocolors for apoptotic lymphocytes, rendering possible the yield of false-positive results for propidium iodine staining. The reported negative correlation between CD3/CD4 cells and the percentage of ANNEXIN-V/CD4/CD3 cells allows one, with safety, to trust the analysis of apoptosis.

Contrary to CD4-positive T lymphocytes, B lymphocytes (that is, CD19-positive cells) remained unaltered (Figure 2). This finding probably suggests that B-lymphopenia, described by others [15], is a phenomenon supervening later in the series of events of sepsis.

The second major early change in the study population was an increase of NK cells (Table 2) directly connected to the outcome of the patients in such a manner that a percentage of NK cells >20% was connected to survival benefit, as opposed to patients with NK cells ≤20% (Figure 1). In a former study by Hotchkiss and colleagues [15], focused on immunophenotyping of cells of the spleen taken from septic patients postmortem or late after the advent of sepsis, an increase of NK cells was described; however, the increase failed to reach statistical significance compared with controls. Our study is the first statistically confirming an early increase of NK cells in severe sepsis. Animal studies of experimental infections by streptococci concluded that triggering of NK cells was accompanied by rapid progression to death [17,18]. The latter experimental findings are opposite to the beneficiary effect of NK cells reported in the present study. A probable explanation might be that in the present cohort of patients severe sepsis was of Gram-negative origin, whereas pathogens were Gram-positive cocci in former animal studies.

An explanation for the protective effect of increased NK cells for the septic host may be provided by the positive correlation between the percentage of NK cells and serum sTREM-1 concentrations. sTREM-1 is a novel mediator considered to play an anti-inflammatory role in sepsis [5]. The present study is in accordance with previous results by other authors connecting serum levels of sTREM-1 >180 pg/ml with prolonged survival of the septic host [11] (Figure 2). NK cells have never been reported to be a reservoir for the production of sTREM-1. *In vitro *results with cells of healthy volunteers revealed the potency of NK cells for the production of sTREM-1 upon triggering with lipopolysaccharide (Figure 3). Moreover, the triggering receptor expressed on myeloid cells-1 has a functional homology to the NKp44 receptor expressed on cell membranes of NK cells [19]. If NK cells of the septic hosts are a source for sTREM-1, then their protective role in early severe sepsis may be explained.

## Conclusion

The present study provided evidence for the first time that early severe sepsis is characterized by CD4-lymphopenia and an increased presence of NK cells, providing a survival benefit for the septic patient at percentages >20%. The survival benefit resulting from elevated NK cells may be connected to elevated serum levels of sTREM-1.

## Key messages

• Early severe sepsis is characterized by CD4-lymphopenia and an increased presence of NK cells.

• Percentages of NK cells in early severe sepsis >20% and concentrations of sTREM-1 in serum >180 pg/ml are accompanied by prolonged survival.

## Abbreviations

ELISA = enzyme-linked immunosorbent assay; NK = natural killer; PBS = phosphate-buffered saline; pO_2_/FiO_2 _= partial oxygen tension to oxygen fraction ratio; sTREM-1 = soluble triggering receptor expressed on myeloid cells-1.

## Competing interests

The authors declare that they have no competing interests.

## Authors' contributions

EJG-B participated in the study design, coordinated the lab job, analyzed the data and wrote the manuscript. TT participated in the study design and the estimation of sTREM-1. ES and VK participated in the immunophenotypic analysis. MM participated in the follow-up of patients. DP participated in the enrolment of patients. IV, AA and VV participated in the follow-up of patients. HG drafted the manuscript.

## Figures and Tables

**Table 1:** Clinical characteristics of 49 patients with early severe sepsis enrolled in the study

Characteristic	Severe sepsis
Age (years)	77.0 ± 11.9
Male/female	28/21
Acute Pathophysiology and Chronic Health Evaluation II score	20.2 ± 9.1
Sequential Organ Failure Assessment score	8.53 ± 3.35
Underlying infection	
Acute pyelonephritis	29 (59.2)
Acute intra-abdominal infection	12 (24.5)
Nosocomial pneumonia	9 (18.4)
Bacterial pathogen	
*Escherichia coli*	10 (20.4)
*Klebsiella pneumoniae*	3 (6.1)
*Acinetobacter baumannii*	3 (6.1)
*Morganella morganii*	2 (4.1)
Others	2 (4.1)
Bacteremia	9 (18.4)
Case fatality	27 (55.1)

Data presented as the mean ± standard deviation or as *n *(%).

**Table 2:** Subpopulations of lymphocytes of 49 patients with early severe sepsis and six healthy controls

	Median	5th percentile	25th percentile	75th percentile	95th percentile	*P *value
CD3/CD4 lymphocytes (%)						
Controls	45.83	39.40	42.24	53.74	-	<0.0001
Severe sepsis	24.71	2.85	13.97	30.27	44.76	
CD3/CD8 lymphocytes (%)						
Controls	24.87	14.92	19.89	28.09	-	NS
Severe sepsis	20.93	3.09	13.00	30.53	51.39	
CD3/CD(16+56) lymphocytes (%)						
Controls	4.50	1.77	1.91	7.43	-	NS
Severe sepsis	6.10	0.76	3.03	10.75	24.75	
CD19 lymphocytes (%)						
Controls	8.74	7.76	7.81	11.62	-	NS
Severe sepsis	4.70	0.48	2.51	10.09	22.77	
CD3(-)/CD(16+56) lymphocytes (NK cells) (%)						
Controls	4.12	0.25	1.26	8.94	-	0.011
Severe sepsis	11.32	2.25	7.05	18.25	33.69	

NS, not significant.
